# Cataphoresis

**Published:** 1898-11

**Authors:** Arthur Holbrook

**Affiliations:** Milwaukee.


AMERICAN JOURNAL

—OF—

DENTAL SCIENCE.

VOL. XXXII. BALTIMORE, NOVEMBER, 1898. No. 7.

### Selections.

ARTICLE I.

### CATAPHORESIS.

ARTHUR HOLBROOK, D. D. S., MILWAUKEE.

Read before Wisconsin State Dental Society, July 20–22, 1897.

The success of all dental operations depends primarily upon thoroughness. Failures of fillings in living teeth can ofter be traced to improper or imperfect preparation of cavities, and on the other hand the success in almost hopeless cases of decay in "dead teeth" is due to the simple reason that the tooth could be properly prepared without pain. The pain inflicted has been a trial to the dentist as well as the patient, and it is safe to assert that not only the happiness but the health of the conscientious dentist is impaired by the terrible strain upon his strength and nervous forces. So the hearts and the brains of dentists have long sought for a means to allay the sufferings of their patients; and it may be added, parenthetically, to the credit of the profession, that the seeking for relief has been upon the part of those who have inflicted the pain and not by those who endured it.

In this paper it is not my purpose to present an original idea or achievement, but to deduct truths from a great array of miscellaneous and often contradictory statements; and by giving the results of a somewhat painstaking personal observation and study to solve for some of you the problems which have arisen with what is perhaps the subject of most vital interest to the dental surgeon to-day—cataphoresis.

Because most dental operations are minor and the prognosis never serious, and because they are prolonged, the common use of a general anesthetic is utterly impracticable. So that with the common use of general anesthesia in surgery, it is a fact that more pain is inflicted upon the people by the dentist than by the surgeon of any other department of science. For a decade article after article has appeared in dental literature presenting this or that form of local anesthesia, while in the advertising pages one could count scores of drugs and appliances which have been offered to secure truth for the quack who advertises his "Painless Dentistry." And it seems now that with our better understanding of drug action, with the development of histology, chemistry and physics, the time must be near at hand when we can apply our local anesthesia as successfully and commonly as does the general surgeon his ether or chloroform.

Of the methods of securing this result cataphoresis is the latest, and from a conscientious study of the subject and a very uniform success in its application, I sincerely feel that, with all its present imperfections and limitations, cataphoresis is capable of a development which will place it in the category of those discoveries and inventions by which the advance of our profession has been measured.

Although new in its application to dentistry, the use of electricity for the promotion of osmosis is old, and ever since Dr. Richardson expressed the theory in 1858, it has been used in medical and surgical practice to hasten and increase drug absorption. After its marked success in these fields it is not surprising that some of our original thinkers should conceive the idea of usidg it to facilitate the introduction of drugs into the hard, dense tissues of the teeth.

There are many theories as to the nature of electricity and its effects, and hence there are many theories as to how electricity promotes osmosis. Some students of cataphoresis go so far as to deny that osmosis of the drug into the tooth occurs, and that the anesthesia is accomplished by paralysis resulting from the current. Of this we shall have more to say later, and for our present needs we have but to accept the fully established fact—that molecular disturbance in a solid caused by an electrical current hastens the absorption of some liquids by that solid in the direction of the applied current. To this end we apply an electrical current in such a manner as to pass from the anode, applied to the tooth, through the tooth, through a part of the body to the cathode, which is most conveniently held in the patient's hand. At the point where the current enters the tooth is placed the drug to be absorbed.

Simple as this may seem, with the carefully arranged and simplified apparatus at our disposal, there are nevertheless a variety of elements that enter into the application of cataphoresis, which are of such importance that the failure of a single one may mean the total loss of effect, or may endanger the life of your patient.

I have read articles and accounts of discussions which show clearly that the men giving their experience with cataphoresis have lacked knowledge of the first principles governing its application. That they have bought their outfits and followed the arbitrary rules of the manufacturer's catalogue or agent, and are now disputing the real students of the subject, or revealing their ignorance by worthless magazine articles or a list of failures which they scoffingly present. The dentist must know his apparatus. There are many cataphoric outfits on the market, and many of them are thoroughly reliable. It is certainly unnecessary to follow the advice which has been given, for the dentist to manufacture most of the apparatus for himself, although he may improve many little details in the yet imperfected appliances.

If the instrument is connected to the street current with proper precautions, it is just as safe and reliable, and certainly cheaper and more satisfactory than the maintenance of office batteries. Of course the dentist must guard against the accidental grounding of the wire from the patient's contact with fountain cuspidor or other possibilities, but this insulation of the patient can be easily and perfectly accomplished. With many instruments the use of the street current is an impossibility, and even the stoppage of an electric car is felt by the patient; but with proper resistance before the current reaches the rheostat —and with a reliable rheostat the variation is positively unnoticeable and the current to all practical uses is unvarying—the change of pressure back of the resistance does not perceptibly influence the flow beyond the rheostat. I am able to quiet the fears of my most nervous patients when I explain that the system I use (from no electric lighting plant) has no overhead wires, and in the almost impossible case of a sudden shock —a crossed wire, a lightning stroke—there are between the outside wire and the patient, on different floors and in different parts of the building, seven separate fuses, any one of which would instantly burn out, as well as numerous lamps, rheostats and other shunts.

Having the instrument connected, it is necessary before application to determine the polarity of the current. How many a failure in the use of cataphoresis has been made by the careless application of the poles, so that the current was running through the body and out of the tooth—absolutely preventing the absorption of the fluid. I personally know of cases where dentists have recorded failure because of this fault, and many of these men who have found success in about every fourth application would raise their average by attending to the polarity. In my practice the attendant tests the polarity before each operation by applying the poles to a solution of iodid of potassium and noting the characteristic discoloration at the anode. Although in 100 cases so tested with my present apparatus the polarity has been nearly constant, I have known it to vary almost daily in other instruments; and, although having a reliable milliamperemeter in the current, I believe the various chances for possible alternations of current demand this repeated test of polarity.

The instrument being adjusted and the polarity known, it is now of the greatest importance that we determine the resistance offered to the current by the patient. Of this resistance, that presented by the body aside from the tooth is of comparatively small variation, while the resistance of the tooth itself may vary, as has been estimated, from five thousand to eighty thousand ohms. This is a point upon which I wish to lay particular stress, for many dentists in using cataphoresis attend merely to the pressure of the current used, with no knowledge whatever of the amount of flow of that current through the objective tooth. They may know the voltage of their current, and by diminishing a certain number of ohms on their rheostat, apply to their satisfaction a certain current strength, but with absolutely no knowledge of the resistance offered by that tooth, how can they intelligently determine the proper current strength, to be used? Gentlemen, let me assure you that the turning on of a certain amount of current, with no reliable knowledge of the resistance offered to that current, is an act of folly, if not of danger. Personally I should never use a cataphoric outfit without a frequently tested milliamperemeter to measure the current which flows through the tooth, thus indirectly measuring the resistance offered by the tooth and giving me a perfectly reliable guide as to the amount proper to apply.

The track traversed by the arm of my rheostat I have divided into tenths for the purpose of noting and recording the minimum pressure to secure a certain flow of current through a tooth—which is shown by comparison with the milliamperemeter. Despite the increase of pressure from behind, a limit of current through the tooth and milliamperemeter is often reached and maintained because of the resistance of the tooth. But it must be remembered a high pressure upon a heavily resisting tooth may be injurious and even dangerous. Twenty volts is considered harmless for any one to take, and to make the current absolutely safe it ought never exceed that amount. To secure this safety the current can easily be regulated by lamp resistance so that it will never measure more than twenty volts even before it reaches the rheostat. The current can be easily and positively reduced through shunts by an expert electrician. With such connections and certain knowledge of the amount of current flowing throuh a tooth, the much discussed danger from using a "110 volt street current" are not to be feared.

Before leaving the subject of adjustment of the apparatus I wish to urge upon you the necessity of keeping the instrument as completely out of the patient's view as is practicable. The temperaments of our patients must be carefully studied, and while we may have to explain in detail to the electrical genius, to the hysterical woman or the frightened child, the less display the more satisfactory our results, and the possible avoidance of a paroxysm that staggers us with fear of too strong a current or of cocain poisoning.

Thus understanding our apparatus, we must acquaint ourselves thoroughly with the proper methods of preparing the field of operation. We must remember the first axiom of electricity—that the current follows always the lines of resistance; and hence make our first object the insulation of the tooth. The moisture which collects between the cavity and the gums, and adjacent metallic appliances or proximal fillings, are the commonest causes for a deflection of current from the solid resisting tooth. The external tooth and margins of the cavities must be carefully tried, while all adjacent metals, whether of fillings or appliances, must be guarded by insulating pads, a layer of wax or equally effective means. Deep-seated cervical and approximal cavities with old fillings, especially in molars, will often be found difficult to isolate.

Our attention should next be given to the drug we employ for the anesthesia. It has been claimed by some that this is of the least importance. It has been said that the usefulness of cataphoresis is limited to its hypnotic power, and that were the element of imagination eliminated, it would be without effect. This so-called psychotherapy, which our faith cures illustrate, is without doubt an element in many cases, but that cataphoresis depends on that little element for its success I am able to dispute point blank. To those who claim that water is as useful an agent to administer as any other I must also protest, for repeated experiments disprove the claim.

Perchance, in addition to the hypnotic element, anesthesia may be aided by more or less paralysis, and although I have never seen such a statement, it seems possible that Schleich's infiltration method may play some part in the deadening of the pain. This latter method, which the surgeon has found so useful in minor operations on soft parts, consists in infiltrating the tissues by hypodermic injections—enormously increasing the tension about the operation field. This has the effect of paralyzing or numbing the sensation of the area, and although accomplished in degree by solution of salt and water, it is far more effective by the use of a local anesthetic in the solution. As this theory has been found practical to the general surgeon, and of late to the oculist, I suggest the possibility of its being an element in dentistry, where solutions are forced with unusual power into the already firm, dense tissue.

Admitting these elements as aids to our anesthesia from the literature on the subject and from personal observations, I submit: that the successful application of cataphoresis is impossible without the agency of a proper solution of cocain or other local anesthetic.

At present there is before us no drug to approach cocain in efficacy for catophoric application. In studying the preparations of cocain I have had considerable correspondence with an expert chemist, whose daily occupation year after year is the testing and superintending of manufacture of the greater part of the cocain used in this country. He says: "Cocain is the most easily decomposed of all alkaloids. Solution in water of the hydrochlorate (its common form) commences to decompose at once, although the process is very slow at first. If the solution is acid in reaction the decomposition is quicker, and heat hastens the process still more." The addition of antiseptics and germicides prevents the formation of molds, but there is no preventive yet discovered for the chemical decomposition. For us who formerly kept our cocain solutions for months, and had the bottle refilled from the druggist's stock bottle, this may explain some failures of applications. I was recently in the operating-room of a large hospital and was shown a row of bottles for hypodermic use; strychnin, digitalis, nitro-glycerin and others, all in solution; but on the end of the row was a bottle marked "cocain;" filled with powders and labeled with an injunction to make a fresh solution when used. So whatever preparation we use, it may be kept in powder, tablet or crystal form, and be applied from a fresh solution.

Since acid hastens decomposition we must secure a neutral or weakly alkaline reaction at the field of operation, and must assure ourselves that bottles, mortars, cotton and anything which comes in contact with the cocain are not soiled with acids.

As to the effect of electrical currents upon the stability of cocain there has been much discussion. I have reserved for this connection what I have to say regarding the strength of current employed; for it is not digressing from the subject now under consideration, but it concerns it most intimately. There have been claims, and by excellent men, that a current strong enough to induce osmosis into a tooth would decompose cocain; by others that a strong current was necessary for the osmosis, and that the electrolytic effect on the cocain was what was necessary to produce anesthesia; still others asserts that the weakest electrical currents cause electrolysis and are not sufficient to cause osmosis. Clinical facts disprove some of these views, and to corroborate the clinical experience and definitely decide the disputed points come the results of experiments under Professor Herdman's direction at Ann Arbor, which are undisputably reliable. The conclusions which he reaches that concern us are, in his own words: "First. That cocaine hydrochlorate solution is cataphoric. Second. That with the strength and density of current ordinarily employed in therapeutic applications there seems to be no decomposition of the cocain hydrochlorate at the anode." And he further concludes that although with these weak currents the intact molecule of cocain hydrochlorate may be transmitted into the tooth tissue, with moderately strong currents the molecule is decomposed. So of what value are these reports from dentists who failed in result, although, as they confidently state, "the current was as strong and painful as the patient could bear."

Gentlemen, do not look for anesthesia where your current causes pain, and put no confidence in the results of the operator who, without a milliamperemeter, gauges his current by the sensations of the patient, or the track of his rheostat. The records of any careful observer will show how weak a current may be effectually employed, how in a hard, dry, pipe-clay tooth almost complete anesthesia may be caused by a current strength of one-twentieth of a milliampere—one twenty-thousandth of an ampere.

If your are chemists enough to care for formulæ of the reactions in the decomposition of cocain, I have them; but aside from the fact of decomposition, the chemical reactions are of no particular interest.

As to the best preparation to employ, we must consider its purity, ready solubility and accurate and convenient division for dosage. For the purity we must depend upon a good druggist who provides us with the article from standard firms. The tests for purity are technical and occupy too much time for us. Most preparations of cocain are readily soluble in water—if we use crystals or tablets they lose no virtue in being triturated and dissolve more readily after such process. The alkaloid itself has been recommended, but as it is insoluble in water and must be used in alcoholic solution of one part cocain to three and a half parts alcohol it is generally undesirable, since alcohol is a poor conductor of electricity. However, this solution of the alkaloid is quite stable, and if absorbed acts quicker than the salts of cocain, so I would suggest the possibility of an advantage from its use in deep cut cavities or directly on the pulp, otherwise it possesses no advantage over the hydrochlorate, which is the most popular form in use and deservedly so. The hydrochlorate is best used in tablet form, which gives a convenient, stable, very soluble and accurately measured dose.

In my practice I am using soluble porous discs of the hydrochlorate, and one is soluble in a drop of water. Many of the tablets upon the market contain in combination more or less extraneous matter to give bulk and consistency, which additions often retard the solubility and effectiveness for dental purposes.

As to the dosage employed, there should be no uncertainty or fear where caution is used. Medical authorities give internally from a quarter to one or even two grains; specialists freely spray and swab the throat with a four per cent solution, representing two and a half grains to the drachm; surgeons ordinarily dissolve from a half to one grain in a hypodermic syringe barrel and inject the entire dose if necessary. I have found a report of a susceptible woman who was made faint, nauseated and depressed by about a quarter of a grain in the nasal passages; and on the other hand of a man who swallowed eighteen grains at one dose without an untoward symptom.

Before deciding on the dosage of cocain for us to employ, let us understand that the object of cataphoresis is not always to secure and maintain a complete anesthesia—local anesthesia is rarely complete, and the general surgeon with his readily accessible soft tissue never expects to perform his operation with a complete loss of sensation. What he strives for, and what we must strive for, is an obtunding of the nerve sensibility to such an extent that the operation is borne in comparative comfort.

The minimum dose of cocain that I can find advocated in the best literature is one-eighth of a grain. I can find no authority who would not inject this dose into an adult with impunity, while the commonly employed dose is between a half and one grain. So when I recommend that a tablet or powder containing one sixth of a grain of the hydrochlorate be triturated and dissolved in a drop or two of water at the time of operation, and this be taken up by a small pledget of cotton and applied to the field, I do so with an absolute certainty that no overdosage will ensue. If every molecule of that cocain were absorbed it could not do the slightest harm, excepting in those extremely rare cases where a remarkable idiosyncracy exists, and never has a case been reported where one-sixth of a grain endangered life. But you can readily appreciate that under the circumstances it would be impossible for the entire dose to be taken. The cotton never absorbs all of the fluid; one-fourth at least is left of the original solution, and on removing the cotton after applying your current there may be squeezed out a considerable part of the dose. At the most exaggerated margin not a third of that sixth of a grain is absorbed, perhaps not a fiftieth. For argument let us say a third is absorbed—a dose of one eighteenth of a grain, which is not enough to harm our youngest or feeblest patient. This dosage of one-sixth grain represents one drop of about a sixteen per cent solution. Experience has proven that this dose is ample, and although seemingly too small to be effective, experiments fully show that its omission or substitution by water is without the anesthetic result.

If by any accident we should apply an overdose to our patient, if our assistant is careless, or our measure faulty, or if the patient swallows a quantity, we should look for these symptoms: faintness, giddiness, nausea, pulse small, rapid and intermittent, pupils dilated, severe prostration, respiration slow and feeble. To meet these conditions the patient should be laid flat and the clothing loosened, while we should employ every means of stimulation at our command. Get the patient thoroughly heated, give hot stimulating drinks, and if necessary perform artificial respiration by slowly raising the arms high above the head for inspiration and bringing them slowly forward and down on the chest with considerable pressure for expiration. Some dentists keep on hand the small pearl beads of amyl nitrite to be broken into a handkerchief, from which the prostrated patient inhales. They are very convenient and are highly recommended for cocain poisoning.

We must, however, differentiate between hysteria and cocain poisoning. In hysteria our patients would not have the intermittent pulse, they would be emotional and solicitous instead of prostrated, and their rapid, strong breathing would take the place of the slow, feeble respirations of the cocain sufferer.

The unfortunate element of time in the application of cataphoresis caters to this fear, for the patient, already wrought to a high tension by approaching operation, is made to quietly sit for a quarter of an hour with this strange, mysterious, electrical instrument applied—lightning strokes, crossed street wires, the electrocution chair are brought to mind, and if the patient be at all susceptible he is under most favorable conditions for a nervous paroxysm. Until the present system be replaced by one that requires shorter application, we must study to divert our patient's attention and to be always reassuring of their safety.

Many patients are solicitous lest some permanent injury to the teeth follow the electrical application, and this fear is in every way reasonable, since it has occupied the attention of some of our leading thinkers. But the apprehensions of these more conservative men, that the long use of the current may injure the pulp, are being less and less expressed, since the value of very weak currents has become better understood and the clinical reports of hundreds of carefully applied and carefully observed cases have shown the harmlessness of the current.

Before we enter into a discussion of this subject I desire to briefly summarize the points I feel to be of vital importance to the dentist in successfully applying cataphoresis : First to understand the fundamental principles of the theory of electrical osmosis. Then to thoroughly know his apparatus; its relations to the current it receives, with especial regard to connection, shunts and resistance, being certain for absolute safety that the current reaching the rheostat never exceeds twenty volts. The rheostat, particularly with reference to the control and steadiness of current. The milliamperemeter, to indirectly measure the resistance of the tooth, and to determine the amount of current. Next, the preparation of the field; its insulation from moisture, fillings and appliances; its proper neutral or alkaline reaction. Then the reagent used : To properly select a preparation ; to use a fresh solution and understand the liability to decomposition and those agents that assist decomposition; to select the proper dosage. Then to appreciate the value of a weak current, since it promotes osmosis and does not decompose the molecule. Finally, to study the temperaments of his patients—after which considerations he should be able to apply cataphoresis in a thoroughly rational and successful manner.—*Dental Digest.*

